# What Will Make Good Teeth?

**Published:** 1872-11

**Authors:** Geo. B. Harriman

**Affiliations:** Boston


### Dental Register.

VOL. XXVI.] NOVEMBER, 1872. No. 11.]

### COMMUNICATIONS.

### What Will Make Good Teeth?

PROF. GEO. B. HARRIMAN, BOSTON.

There may be skill in the dentist in repairing the waste of nature or in the supply of its loss, but if this completes his accomplishments, his usefulness is shortened and his success abridged. A leading aim in the profession should be to produce general conviction that this waste and loss are needless, and consequently shameful and criminal. A reform might thus be inaugurated which would remove the abuses to which nature has been subjected in some instances through ignorance, and in others through the gratification of appetite, that she might in the production of human teeth be in harmony with her other works. The grass comes forth a lovely green, the trees send out healthy boughs and symmetrical leaves, and the flowers are both regular and beautiful as they blossom.

Animals of the lower order ordinarily have no blemish in limb or teeth, and the men of the forest as well as those not pampered with luxurious living are usually free from any dental distortions or dental pains, and show when they eat or laugh the strength and beauty of teeth formed in harmony with nature's laws. Hence instruction to the dentist is of the most important character. It is his province to labor for the restoration of the period when a set of artificial teeth will be as rare and noticeable and excite as much pity and commiseration as an artificial arm or leg now does.

The loss of a single tooth or an entire set is the loss of so much of the individual, and impairs the physical capacity for the discharge of the duties and responsibilities of life. Could it be written over the dentist's office in letters of gold and set in pictures of silver, "*Teeth preserved and not extracted here*," for the reason that success had crowned persevering efforts made to reach this most desirable end, honor, second to no other, would accrue both to the office and profession; of course such a result can not be inaugurated and completed in a generation, but the humane and honorable work can be commenced in our day.

Though the man or men on such a vast reform may but begin it and give its outlines, on their memory, when others after them should take up their work and complete it, would rest the blessings of thousands who were ready to perish. They will be recognized as the fathers of the reform, to prevent unborn generation from enduring the horrors of diseased teeth with the screeching pains of their repair or removal, and the annoying inconvenience of wearing artificial teeth, and last but not least of being saved from the many diseases superinduced by the impurities of the mouth produced by decayed teeth. How can this vast and most benevolent reform be inaugurated? Shall it be in our day? It may be; and who will go forward? It must be commenced in the family: what dentist will make it a specialty among the families of his patients? He can begin with the parents who bring their children to his operating chair, and show that this call for the interposition of the dental art might have been prevented had physiological laws been obeyed. The Framer of the human body made teeth upright, strong and durable, with all materials in the system for their preservation as made, but man has sought out many inventions by which these useful and beautiful members have been abused, shorn of their lustre and made the seat of most excruciating torture. These inventions have been multiplied, not in the drawing or sitting room; but in the room in which culinary operations are performed. Here are contrived ways and means for the destruction of the teeth in the preparation of food which subtracts from bone its enduring properties and makes it soft and brittle, fitting it for easy and early waste and decay. Were the inventions of the cooking room personified, with their baneful effects on the human system accurately drawn out and delineated, a monster would appear such as no eye could behold without terrible fright and no hand touch without fear of deadly poison. To startle his patients with the sight of this monster should that dentist labor who would advance the reform it is our object in the present writing to encourage. Parents might hence be startled to the instant necessity of abandoning those culinary inventions which most essentially and surely interfere with the working of nature in bringing into the human fabric what slowly but certainly destroys.

If such abandonment obtains, then the table around which they and their children tri-daily gather will be furnished "according to law," which law, being in harmony with the demands of healthy nature, would prohibit and abolish all fancy diet and introduce that which would nourish bone and muscle. A table furnished according to correct physiological laws would be destitute of superfine flour and a large supply of substances, and be supplied with bread of coarser material, with husk and bran, the life of bone included, and not "sifted out." Should this reform prevail, though some present good would result to the families adopting it, coming generations faithfully practicing it would show the ulterior and real benefits, not only by teeth lustrous with beauty artistic with uniformity, and strong and massive, but in a general robust frame and continuous health, strength and vigor. After this obedience to physiological laws by a few generations, children would be born who would seldom if ever have necessity for approaching a dentist's chair. Then the use of every member of the human frame would be known and every member would be *used* according to *design*. The mother having the requisite, yea, even more than the requisite supply of phosphates, would bring forth her child well constituted for timely development. The teeth would early erupt and with regularity. They would advance to maturity with strength, and if the diet was correct, would *abide* and as a rule be free from disease and pain. In this halcyon period, when what is written above will be the experience, the teeth will be *used.* Now they are not. Food is so prepared and often so furnished in kind that all the teeth are not subjected to their necessary and appropriate employment in mastication. Mastication of appropriate aliment is indispensable to firmness and resistance to sound and durable teeth.

Should the dentists of our country hold the views expressed in this article and practice accordingly, we might soon gain for our profession a character more illustrious than victory on the battle field, and more enduring than if chiseled on marble; for our monument would appear in generations not yet born, and would not disappear until the last of the human family had been entombed. To be thus useful and to gain this great distinction our duty is first to become familar with the component parts of the human system and the nutriment necessary to supply and sustain each and every part, to make it sound and durable.

The composition of the human body is as follows: oxygen, hydrogen, nitrogen, carborn, phosphorus, calcium, fluorine, chlorine, sodium, iron, potassium, magnesium, silicon and sulphur, making fourteen elements to be supplied every day that the system may be in its appropriate and healthy condition. The source of this supply is the food taken into the system, the air we inhale, and water. The food can be divided into three classes. First, That which supplies the lungs with fuel and thus furnishes heat to the system and supplies fat. This we will denominate carbonaceous or heat-producing food, carbon being the principal ingredient. That which supplies the greater part of the muscle we will denominate nitrogneous; nitrogen being the constituent principle and that which supplies bone, teeth, brains and nerves, and imparts vital power to muscular and intellectual activity, we denominate phosphatic, phosphorus being the main element. The waste, and consequently the supply, of these three classes of elements is very diverse; four times as much starch and fat or carbonaceous food being required as nitrogenous, albumen and casein; whilst of phosphatic not more than two per cent. of the carbonaceous and heat-producing substance is demanded. The loss of these elements in the man of moderate size will be about one pound per day. Food, therefore, must supply these elements. In animal food the carbonacious is furnished in fat, the nitrogenous in fibrin, casein and albumen. In vegetable food the carbonacious is supplied in sugar fibrin and casein. In the elements of the body we find there are fourteen, and not any of them, except the enamel of the teeth, are perfectly fixed in the system; but each after a short performance of duty assigned it by nature becomes effete or waste substance, and is supplanted by other particles of the same nature, which of course are supplied in food. Each organ requires a different substance, and has the power of abstracting from the mass of elements circulating in the blood what may be necessary for renewing its vitality and of rejecting what is not appropriate to this end. While these fourteen elements having existence in plants or vegetables are supplied as wanted, peace and harmony are dominant in the system, producing perfect health; but the introduction of any other element into the circulation would create disturbance and excite the other organs to labor for the expulsion of the intruder, and during this conflict there would necessarily be disease. We hence find the foundation for a scientific adaptation of food to the different vocations of life. The man who works in an atmosphere at zero, and the dentist at his operating chair with the temperature at seventy degrees, consume very different elements, and in different proportions, and therefore the two demand different elements in their food. The one requires a large supply of the nitrogenous and carbonaceous elements for the supply of heat and muscle, whilst the other requires but very little of these, his need being the phosphatic, that the brain may maintain its power. The all-wise Creator has anticipated the necessities of diverse conditions, and has made provision whereby they can each be met and satisfied in different kinds of food, and therefore individuals in various avocations can have what they need for their best health and vigor. By carefully looking after and strictly following the laws regulating their being, as has been before intimated, human beings would have the reward in their own health. The blessing in greater richness would descend to their children, who would in a few generations be possesssed of the firmest health and the soundest teeth, with a disposition for kindness and affection greatly improved.

It is an old and common saying that bread is the staff of life. The maxim must have originated many generations back, when bolting mills were unknown and seives but little used, when bread was made with all the ingredients of the grain included. Such bread is the staff of life. The bread of our time is, most of it, the handmaid of disease. If there is in it any staff. it is weak and brittle, which will soon let a man down. Wheat more than any other grain, perhaps, contains all the elements needful for the nutrition of the system. To have this effect, however, all its properties must enter the bread made of it; none must be bolted or sifted out, nor must the stone grind it too fine.

In the analysis of wheat we discover water fourteen parts, nitrogenous or muscle-producing material little more than this quantity, carbonaceous heat and fat-producing materials a slight advance over sixty-nine parts, phosphatic for teeth, bones and brains, two and a half parts. The three principal substances entering the fourteen elements which constitute the human system and the proportions of the muscle-making, teeth, bone and heat-feeding elements, are about the average proportions demanded with moderate health, and in moderate exercise of the mental faculties with moderate physical activity.

If we examine a kernel of wheat under the microscope, we shall detect the chip or germ occupying about two per cent., nitrogenous fifteen per cent., carbonaceous (starch-fat) occupying the center.

In closing this article I beg leave to submit to the serious consideration of the profession that it is our duty to be instructors to the heads of families who, with their children, are our patients in relation to the matters herein treated. When we discover children's teeth defective and decaying we ought most earnestly to exhibit both the cause and the remedy and urge instant and continued attention to our counsels and instructions. We are bound to recommend the use of proper food, such as oat meal, barley, unbolted wheat, peas, beans and beefsteak, and other food designed to make strong bone, good teeth and healthy bodies. In acting according to our obligations we shall be rendering a service of vast benefit to our race.

